# Differential Gene Expression and Aging

**DOI:** 10.1100/tsw.2002.135

**Published:** 2002-03-09

**Authors:** Laurent Seroude

**Affiliations:** Department of Biology, Biosciences Complex, Queen’s University, Kingston, Ontario, K7L 3N6, Canada

**Keywords:** senescence, gene expression regulation

## Abstract

It has been established that an intricate program of gene expression controls progression through the different stages in development. The equally complex biological phenomenon known as aging is genetically determined and environmentally modulated. This review focuses on the genetic component of aging, with a special emphasis on differential gene expression. At least two genetic pathways regulating organism longevity act by modifying gene expression. Many genes are also subjected to age-dependent transcriptional regulation. Some age-related gene expression changes are prevented by caloric restriction, the most robust intervention that slows down the aging process. Manipulating the expression of some age-regulated genes can extend an organism's life span. Remarkably, the activity of many transcription regulatory elements is linked to physiological age as opposed to chronological age, indicating that orderly and tightly controlled regulatory pathways are active during aging.

## INTRODUCTION

Aging is a universal biological reality that is familiar to everyone. Although one can easily distinguish what is young and what is old, defining aging itself is still subject to controversy. The definition proposed by Robert Arking is one of the most global and rigorous descriptions of the aging process: "the time-independent series of *cumulative, intrinsic, and deleterious functional and structural changes* that usually begin to manifest themselves at reproductive maturity and eventually culminate in death"[1]. Despite being a universal process, aging is limitless in its variety[2]. Obvious differences in aging exist among species and individuals, and even among the various organs, tissues, and cells within an individual. While environmental conditions play a large role, in both humans and invertebrates genes also determine how an individual will age and how long it will live[3,4,5,6].

The regulation of the developmental process that allows a single cell to become a complex organism capable of procreation is ultimately controlled by genes. It is now well known that a very precise regulation of the expression and combinatorial action of many genes differentiates humans from chimpanzees, which have less than 5% differences at the genetic level. Is it possible that subtle changes in gene expression could result in similarly large changes in aging? This seems quite plausible when one considers the famous quotation of Nobel Laureate Francois Jacob: "It is truly amazing that a complex organism, formed through an extraordinarily intricate process of morphogenesis, should be unable to perform the much simpler task of merely maintaining what already exists." Although aging may be characterized by the loss of some kinds of homeostasis, it is not associated with a pervasive decline of regulation of gene expression. Gene expression appears to be well regulated even at older ages when biological performance is generally diminished[7,8]. In contrast to the evolutionary idea that aging is the detritus of the absence of selection after the reproductive period and just represents the organism falling apart, studies of gene expression have shown that aging is associated with the same highly dynamic regulated changes observed during development.

## GENETIC PATHWAYS INFLUENCING LONGEVITY

Laboratory strains of flies have been successfully bred to generate strains with increased longevity[9,10,11,12,13,14,15]. Independently created long-lived *Drosophila* differ in a number of aspects of their physiology[16,17], emphasizing that there are a multitude of aging processes and that life may be extended significantly by activating or suppressing different combinations of mechanisms. Genome-wide genetic analysis has also led to the identification of chromosomal regions associated with the impressive longevity of human centenarians[18]. Despite their success in demonstrating that genes influence aging, selective breeding and genome-wide analysis are macroscopic approaches that cannot easily be used to identify longevity regulating genes. Genetic screens for single-gene mutations affecting longevity have efficiently revealed such genes. Single-gene mutants with extended viability have been found in the nematode *Caenorhabditis elegans*[19,20,21,22,23,24,25,26,27,28], the fruit fly *Drosophila melanogaster*[29,30,31,32], the yeast *Saccharomyces cerevisiae*[33,34,35], and the mouse *Mus musculus*[36]. The molecular analysis of several of these mutants identified genetic pathways implicated in life-span regulation that are strongly suspected to mediate their effect through the regulation of gene expression.

### The Insulin/DAF-2 Pathway

*C. elegans* mutants with a reduced level of the insulin/IGF1 receptor homologue DAF-2 live more than twice as long as wild-type[23,37]; for review[38,39,40]. Manipulation of the amount of insulin/IGF1–like proteins that may act as DAF-2 ligand also affects the life span[41,42]. DAF-2 activates the phosphatidylinositol-3-OH kinase (PI[3]K)/3-phosphoinositide-dependent kinase-1 (PDK1)/Akt signal transduction pathway[43,44,45,46,47]. As expected, *age-1* (encoding PI[3]K) and *pdk-1* (encoding PDK1) mutants with a decreased activity in components of this pathway also live longer[19,20,44,46,48]. Inhibitors of the PI(3)K are also found to mimic the effect of *age-1* and *pdk-1* mutations [49]. DAF-18, a PTEN phosphatase homologue, can counteract the effects of AGE-1 on the phosphatidylinositol-phosphate molecules, and it dramatically reduces worm longevity when mutated[45,46,50]. The life-span extension caused by mutations in components of the insulin/DAF-2 pathway requires the activity of the gene *daf-16*[51,52], which encodes a member of the hepatocyte nuclear factor 3 (HNF-3)/forkhead family of transcriptional regulators.

*In vitro*, AKT phosphorylation of DAF-16 prevents the binding to one of its target sites, the insulin-like growth factor binding protein-1-insulin response element[53]. In cultured cells, insulin signaling to PI 3-kinase/AKT inhibits the ability of a GAL4 DNA binding domain/DAF-

16 fusion protein to activate the insulin response element, but does not affect GAL4 DNA binding site, which suggests that insulin inhibits the interaction of DAF-16 with its cognate DNA site[53]. *In vivo*, disrupting Akt-consensus phosphorylation sites in DAF-16 causes nuclear accumulation in wild-type animals, suggesting that the insulin/DAF-2 pathway prevents DAF-16 accumulation in nuclei[54]. However, forcing DAF-16 to the nucleus has little effect on life span, which indicates that the insulin/DAF-2 pathway must have additional targets to regulate longevity[54]. Mutations in *daf-12* do not extend adult life span, but certain combinations of *daf-2* and *daf-12* mutant alleles nearly quadruple it, demonstrating that insulin/DAF-2 signaling pathway interacts with the DAF-12 pathway to regulate adult longevity[55]. DAF-12 is a nuclear receptor containing a DNA-binding domain[56,57], and a transducer of hormonal signals to the nuclei, where it regulates the expression of target genes.

Mutants in components of the insulin pathway have also been found recently to affect longevity in other organisms. *D. melanogaster*, like vertebrates, use an insulin-receptor substrate (IRS) protein to couple receptor activation and PI(3)K signaling. Chico (encoding IRS) mutants live almost 50% longer than the wild type[31]. Animals heterozygous for mutations in the insulin/IGF-1 receptor live 85% longer than normal[32]. In *S. cerevisiae*, mutation in SCH9 (which is homologous to *Akt*) increases resistance to oxidants and extends life span by up to threefold. Stress-resistance transcription factors MSN2/MSN4 were required for this life-span extension[58]. Taken together, all these observations strongly suggest that the regulation of longevity by the insulin/DAF-12 pathway is mediated by differential gene expression, at least partially. The genes targeted by this pathway and the processes affected remain to be elucidated.

### Silent Regulator Pathway

Aging studies in *S. cerevisiae* identified a gene involved in the silencing of chromatin; for review[38,59,60,61,62]. Silencing is a mechanism that can regulate large chromosomal regions by making them transcriptionally inactive. This kind of regulation requires the activity of a complex of silent regulator (SIR) proteins that include SIR2, SIR3, and SIR4[63]. Deletions of the *Sir2*, *Sir3*, or *Sir4* genes all shorten the life span[64]. Sir4 mutations modifying the chromosomal localization of the protein were found to extend yeast life span[34,65]. Overexpression of the SIR2 protein also extends the life span and is associated with a global deacetylation of histones, a critical reaction that changes the three-dimensional structure of a region in the chromosome, making it inaccessible to the transcription machinery[64,66]. Overexpression of SIR2 has also been found to increase *C. elegans* life span[67]. The SIR2 protein has been biochemically characterized as an NAD-dependent histone deacetylase[68]. All of these observations strongly suggest that silencing, and indirectly modification of gene expression, is an important regulatory component of the life span.

## AGE-RELATED DIFFERENTIAL GENE EXPRESSION

The insulin/DAF-2 and silent regulator pathways output is clearly linked to the regulation of gene expression. Many changes in gene expression have been observed during aging in many organisms, from unicellular yeast to primates[33,69,70,71,72,73,74,75,76,77,78,79,80,81,82,83,84,85]. Differential screening of cDNA libraries isolated from young and old mice identified genes in which protein products are involved in many different cellular processes. One study identified a major urinary protein MUP2, the immunity-related protein Q10 from the major histocompatibility complex (MHC), a cytoskeletal actin, and a creatine kinase[86]. It is worth noting that age-related changes in muscle actin have also been reported in *D. melanogaster*[80]. It is tempting to speculate that this might be related to age-associated deterioration of muscle functions. Another study found a mRNA, with increased abundance in old rat liver, encoding T-kininogen, and induced by inflammation[87]. Immunity is another process greatly affected by aging.

**TABLE 1**
**Function of 12 C. *elegans* Age-Regulated Genes**

| Clone | Change | Function |
|---|---|---|
| Y1 | Increase to mid-life then decrease | Unknown |
| Y6 | Decrease | Vitellogenin 6 |
| Y7 | Decrease | Vitellogenin 5 |
| Y10 | Increase to mid-life then decrease | Not analyzed |
| Y15 | Decrease | Vitellogenin 2 |
| Y37 | Decrease | Unknown |
| Y40 | Decrease | Unknown |
| Y41 | Decrease | Not analyzed |
| Y42 | Decrease, absent in old | Not analyzed |
| O3 | Increase to mid-life then decrease | Unknown |
| O42 | Increase | Unknown |
| NC1 | Increase | Translation factor EF-1α |

Adapted from Fabian, T.J. and Johnson, T.E. (1995) Identification genes that are differentially expressed during aging in *Caenorhabditis elegans*. *J. Gerontol. A. Biol. Sci. Med. Sci.* **50,** B245–B253.

Similar studies were conducted in yeast and nematode. In *S. cerevisiae* these studies led to the identification of the transmembrane proteins LAG1 and LAG2. These proteins are of unknown function but modify the life span when mutated[33,35,85]. In *C. elegans*, 12 genes were identified with different age-related expression changes; molecular characterization was done for 9 of them (see Table 1)[84]. Three of them appeared to be specific to the adult stage (Y6, Y7, and Y15); they also decreased with age and encoded vitellogenin proteins present in the intestine. This might contribute to age-related deterioration of the digestive system. The case of the age-increasing translation factor *EF-1α* gene requires caution, because the opposite observation has been reported in *D. melanogaster*. In one study, a general decline of protein synthesis with age is preceded by a decrease of *EF-1α* mRNAs[88,89]; another study found no significant mRNA change, but did discover a slight drop in the level of the protein and a large decrease in catalytic activity[90]. However, the involvement of EF1-α in life-span regulation has been ruled out by the observation that the overexpression of *EF-1α* does not extend longevity[90,91]. Therefore it is likely that *EF-1α* expression during aging is the consequence of species-specific effects, genetic background, or epi-genetic mechanisms.

Differential display techniques were also used to survey gene expression during aging of the rat brain, heart, and liver[77,81]. The results in the brain identified age-related changes in the expression of the gene *fos* encoding one of the monomers of the dimeric AP1 transcription factor. Further analysis demonstrated that the level of *fos* expression decreased significantly during aging. In contrast, that of *jun*, encoding another component of AP1, increased between 6 and 13 months of age and remained constant thereafter. This results in an age-dependent change in the FOS/JUN ratio that has important implications for the composition of the AP-1 transcription factor as a function of age, and consequently might alter the expression of many genes regulated by AP1[92]. Aging is associated with a decline of brain functions and a high incidence of neurodegenerative diseases. Using immunohistochemistry in rats, differential expression of genes encoding neuropeptides, neurofilaments, and neurotrophin receptors has been reported[78,93,94].

There is an age-related decline in maintenance energy requirement, probably because of a reduction in physical activity and a decrease in basal metabolic rate, which is largely driven by changes in body composition. There is a significant loss of lean body mass and a concomitant increase in fat mass with advancing age. Using Northern and Dot blots on various mouse organs

**TABLE 2**
**Differential Enzyme mRNA Levels Between Young (7 months old) and Old (28 months old) Mice**

| | Liver | | Muscle | | Kidney | |
|---|---|---|---|---|---|---|
| **Enzyme mRNA** | **Young** | **Old** | **Young** | **Old** | **Young** | **Old** |
| PK Glycolytic | 2.5±0.5 | 4±0.5[CR] | nd | nd | nd | nd |
| F-1,6-P2ase Gluconeogenic | 1.67±0.25 | 2.29±0.16 | nd | nd | nd | nd |
| G-6-Pase Gluconeogenic | 10.5±2.5 | 5.5±2[CR] | nd | nd | 18.20±4.96 | 9.94±1.71[CR] |
| PEPCK Gluconeogenic | 1.6±0.2 | 1.1±0.1[CR] | 0.3±0.06 | 0.2±0.02[CR] | 12.79±2.73 | 8.99±1.22[CR] |
| GS Nitrogen metabolism | 6.2±0.8 | 4±0.8[CR] | 3.7±1.2 | 1.5±0.4[CR] | nd | nd |
| CPSI Nitrogen metabolism | 3.4±0.5 | 2.5±0.4[CR] | nd | nd | nd | nd |
| TAT Nitrogen metabolism | 8.5±2 | 4.5±1[CR] | nd | nd | nd | nd |

*Note:* The metabolic function for each enzyme is mentioned.

[CR] Caloric restriction either prevents the level change in the old or maintains old level to a value equal or higher to the value observed in the young not subjected to the restriction.

Adapted from Dhahbi, J.M., Mote, P.L., Wingo, J., Tillman, J.B., Walford, R.L., and Spindler, S.R. (1999) Calories and aging alter gene expression for gluconeogenic, glycolytic, and nitrogen-metabolizing enzymes. *Am. J. Physiol.-Endo. Metab.* **40,** E352–E360.

(liver, muscles, and kidney), age-related changes were reported for the mRNAs encoding metabolic enzymes gluconeogenesis and glycolysis (see Table 2): Glucose-6-phosphatase (G-6-Pase) and Phosphoenol-pyruvate Carboxykinase (PEPCK) decrease, while Fructose-1,6-biphosphatase (F-1,6-P2ase) and Pyruvate kinase (PK) increase[79]. Age-related decreases were also observed for mRNA encoding enzymes involved in the nitrogen metabolism: Glutamine synthetase (GS), Carbamyl-phosphate synthetase I (CPSI), and Tyrosine aminotransferase (TA). In most cases, changes in the mRNA level are correlated with changes in enzymatic activity. Metabolism requires the function of mitochondrial enzymes involved in the respiratory chain. In old flies, the activity of cytochrome oxydase c is reduced in correlation with the level of its mRNA[95]. Additionally the levels of other mitochondrial transcripts are also reduced[96,97]. This almost certainly leads to mitochondria dysfunction that could seriously contribute to the deteriorative changes observed during aging.

All of these studies have revealed changes in all kinds of genes in all kinds of tissues that affect all kinds of biological processes. Because each of these observations were made independently by many investigators, it would be quite tedious and difficult to collect and assemble the data to try to see the big picture of differential gene expression associated with aging. Recently developed microarray technology has made it possible to analyze the transcriptional response to the aging process at a genome-wide level (see Table 3). Such analysis of the mouse skeletal muscle shows increased expression of genes encoding stress-response factors and neuronal factors and decreased expression of genes related to energy metabolism[70]. There is a loss of motor neurons as skeletal muscles age, which triggers the remaining neural units to reinnervate the muscle fibers[98]. The increase in neuronal factors probably reflects this

**TABLE 3**
**Genome-Wide Survey of Age-Related Differential Expression**

| | Up Regulated | Down Regulated | References |
|---|---|---|---|
| **Mouse skeletal muscle** | Stress response<br>Heat shock factors<br>DNA damage factors<br>Oxydative stress inducible<br><br>Neuronal factors<br>Reinnervation<br>Neurite extension | Energy metabolism<br>Glycolysis<br>Mitochondria components<br>Protein turnover | 70 |
| **Mouse brain** | Stress response<br>Heat shock factors<br>Lysosome proteases<br>Oxydative stress inducible<br><br>Inflammatory response<br>Complement cascade<br>MHC molecules<br>Microglial activation factors<br>Inflammatory peptides | Energy metabolism<br>Protein turnover<br>Gluconeogenesis<br><br>Neuronal factors<br>Neuronal development<br>Trophic factor<br>Growth factor<br><br>Stress response<br>DNA damage factors | 73 |
| **Monkey skeletal muscle** | Stress response<br>Heat shock factors<br>Oxydative stress inducible<br>Detoxification<br>DNA damage factors<br><br>Inflammatory response<br>B cell function<br>Inflammatory peptides<br><br>Neuronal factors<br>Neuronal development | Energy metabolism<br>Citric acid cycle<br>Mitochondria respiratory chain<br>Gluconeogenesis<br><br>Growth<br>Cell growth<br>Cell cycle<br>Cell proliferation<br><br>Stress response<br>DNA damage factors | 71 |
| **Drosophila Whole body** | Stress response<br>Oxydative stress inducible<br>Detoxification<br><br>Unknown function | Reproduction<br>Gametogenesis<br><br>Energy metabolism<br>Glycolysis<br>Citric acid cycle<br>Mitochondria respiratory chain<br><br>Stress response<br>Heat shock factors | 69 |

phenomenon and therefore represents a consequence of the aging process. In the brain, in addition to the stress response, genes related to inflammation are also up regulated and the authors suggest that it could be relevant to the progression of Alzheimer's disease and its association with the elderly[73].

## RELATIONSHIP BETWEEN AGING AND DIFFERENTIAL GENE EXPRESSION

Many genes have significant age-specific changes in their RNA levels with increasing age, but most of these changes probably have no causative link with aging. What is the evidence that some differential gene expression could be a cause of aging? LAG2 is preferentially expressed in the young yeast cell. Removing or overexpressing this gene respectively shortens or increases the organism's life span, supporting the hypothesis that the expression change is a factor in aging[35]. Most of the changes observed in the mouse metabolic genes described previously were found to be prevented by caloric restriction, a treatment well known to slow down aging[79,99]. The same observation was made in the large-scale survey of the mouse muscle and brain[70,72,73]. More convincing evidence is coming from the experiments conducted in *D. melanogaster* that use the enhancer-trap technique. This genetic technique allows the experimenter to monitor the transcriptional activity of many regions of the genome (Fig. 1)[100]. This kind of analysis can be used to examine spatial and temporal changes in gene expression down to the regulatory elements that regulate these changes, and therefore to dissect the transcriptional regulatory pathways associated with the aging process. Using this approach, Helfand et al.[101] monitored 49 different regions of *D. melanogaster* genome and found that 80% of them change with age, 55% increasing of which 34% are not expressed at eclosion. Remarkably, the alteration of an individual's longevity, by temperature or mutation, does not affect the pattern of changes but does affect the rate at which they occur[101,102]. Similar observations were made using enhancer-reporter gene constructs in the fly and nematode[103,104]. This demonstrates that gene regulation is associated with physiological age as opposed to chronological age, indicating the existence of a linkage between transcriptional activity and rate of aging.

Apparently an orderly and well-controlled gene expression progression takes place during aging. This raises the question of whether gene expression directs the aging process or whether aging causes changes in expression. With our current knowledge, the second alternative is more probable. Still puzzled by Francois Jacob's statement, I predict the existence of an aging genetic program that has evolved to help the organism to fight the consequences of aging. One approach to testing this prediction would be to manipulate gene expression during aging and examine the consequences. This should be accomplished without altering gene expression at any time during development since this could yield differently assembled individuals, thus comparing apples and oranges. Two conditional expression systems recently described fulfill these requirements. The first system uses a chimeric transcription factor (Gene-Switch) made of the yeast GAL4 DNA binding domain, the human progesterone receptor ligand binding domain, and the activation domain of the human protein p65[105,106]. In the presence of the antiprogestin, RU486, Gene-Switch locates to the nucleus and activates GAL4 dependent promoters. Although RU486 feeding does not induce adult lethality or behavior defects[106], the absence of effects on the life span remains to be confirmed before this system can be applied to the manipulation of gene expression during aging. The second system is based on tetracycline-regulated transactivators “tet-on”[107,108,109] and “tet-off”[110,111,112] that activate tetracycline operator (tetO)–dependent promoters in the presence or absence of tetracycline or derivatives, respectively. Since the derivative doxycycline has been shown to not affect the life span[107], the tetracycline system has already been used successfully to alter specifically gene expression during aging in order to find genes affecting the life span[113].

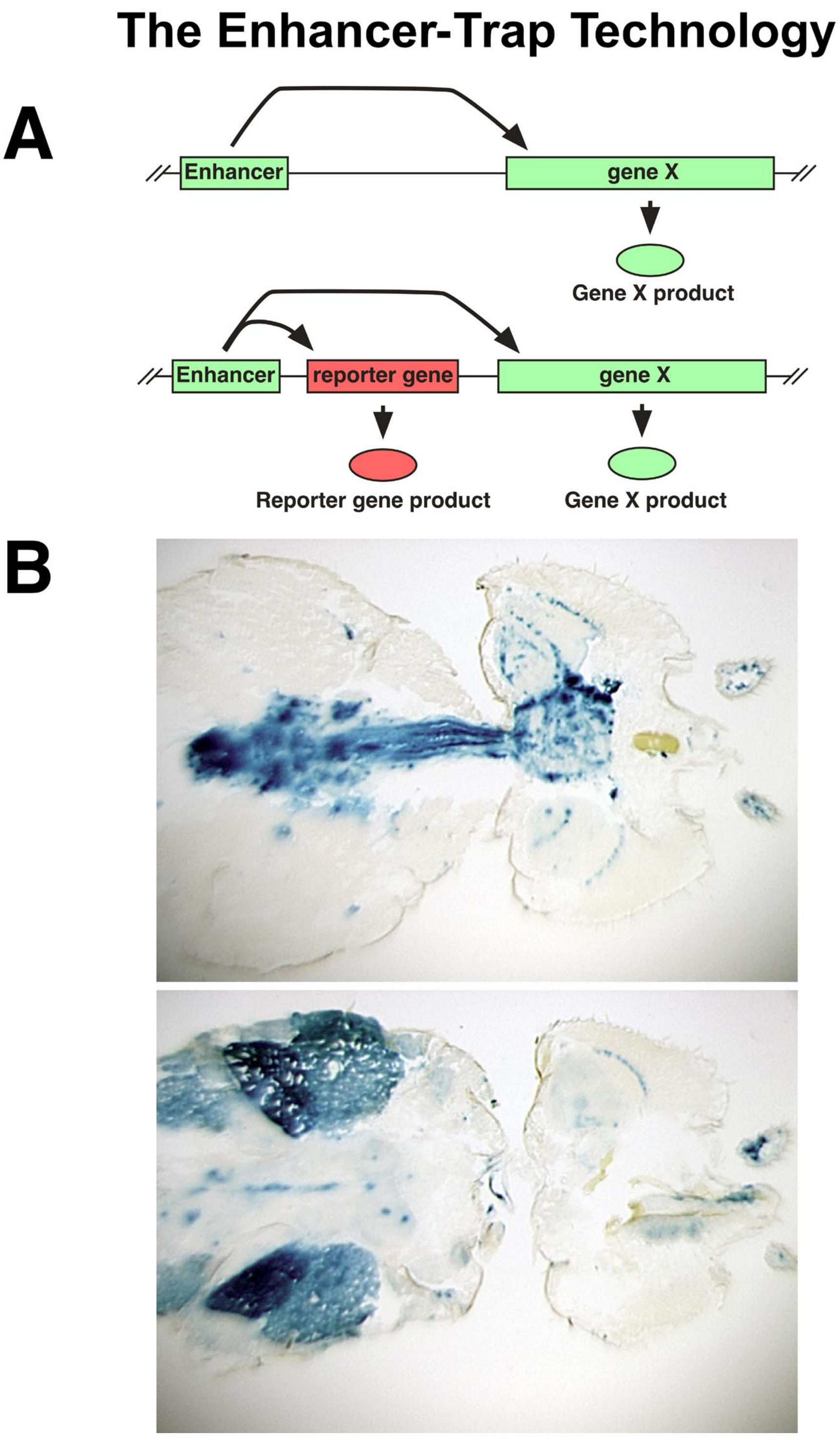

**FIGURE 1.** The enhancer-trap technique. (A) Somewhere in the genome, the expression of the gene X is controlled by a regulatory element called the *enhancer*. In the enhancer-trap technique an engineered piece of DNA that includes a reporter gene is inserted near the gene X enhancer. As a consequence, the enhancer also controls the reporter gene. The reporter gene product can be measured to provide quantitative data about the enhancer activity. (B) The reporter gene product can also be revealed *in situ* to reveal the tissue-specificity of the enhancer. The figures show two enhancers with different tissue specificity at 50 days old (top, nervous system; bottom, muscles). From L. Seroude, unpublished.

## CONCLUSION

Since differential gene expression can affect any biological process, the question remains open as to which gene or which combinations could be manipulated to effectively optimize human vitality and longevity. A multitude of studies have revealed several possible processes to target, such as metabolism and reproduction.

Metabolism is clearly involved because of the correlation between metabolic alterations and longevity and the extension of life span observed in animals subjected to caloric restriction[114,115,116,117,118]. Interestingly, in *S. cerevisiae* caloric restriction requires the activity of *Sir2* gene to increase the life span[119]. This suggest that caloric restriction acts through gene regulation, yielding hopes that we could mimic this effect without having to subject ourselves to severe food restriction. The metabolic rate theory of aging is not enough to explain the process because there are exceptions such as bats, which have a higher metabolic rate than mice, yet live ten times longer. In addition, the metabolic rate of a long-lived insulin receptor mutant fly is normal[32]. Free radicals are produced by metabolism and widely accepted as a better universal candidate for one of the cause of aging[118,120,121]. There is a positive correlation between longevity and the cellular response to stress[122,123]. Long-lived *methuselah* flies are resistant to the free radical–generating agent paraquat[29], and $p66^{shc}$ mutant mice live longer by influencing how cells resist oxidative stress[36]. Several experiments have shown that the overexpression of free-radical scavenger molecules such as superoxide dismutase and catalase increases the life span[124,125,126]. In addition, *daf-2* and *age-1* mutants are resistant to oxidative stress-inducing agents[127] and require the activity of the cytosolic catalase CTL-1 to be stress-resistent and live longer[128]. Importantly, life-span extension is correlated by the up regulation of the *ctl-1* gene, and *daf-2* mutants were also found to have an elevated expression of the Mn-superoxide dismutase *sod-3* gene[129]. Interestingly, a *daf-16* family protein-binding element (DBE) is found upstream of the first exon of *sod-3*[130]. These observations suggest that the insulin/DAF2 pathway affects longevity by regulating genes related to oxidative stress defenses. Recently, superoxide dismutase/catalase mimetics have been described to extend *C. elegans* life span, opening hopes to develop antiaging drugs that enhance the ability of cells to fight free radicals[131].

Reproduction, especially the interaction between somatic and germinal cells, is another process that influences longevity. This is clearly demonstrated by evolutionary biology experimenters who have been able to generate long-lived flies by selecting for late reproduction[9,11]. Other evidence is provided by experiments done on Mediterranean flies[132]. These flies can have two modes of living: a waiting mode in which both mortality and reproduction are low, and a reproductive mode in which these two parameters are high. Flies that switch from waiting to reproductive mode survive longer than those kept in either mode exclusively. These studies support the existence of a trade off between longevity and reproduction, which is not very good news if extending our longevity means preventing reproduction. Cell ablation studies in *C. elegans* support the existence of signals sent by germinal cells, which are important determinants of somatic longevity[133]. Reception of these signals requires the activity of the DAF-16 transcription factor and DAF-12 nuclear receptor but is independent of the DAF-2 receptor, suggesting that these signals influence longevity by regulating a set of genes that are independent of the insulin/DAF2 pathway. The dissection of these signals, and the genes involved in receiving them, might allow longevity to be manipulated without affecting reproduction. Encouraging results are also coming from several fly mutant strains that can live longer without exhibiting a reproductive trade-off (Seroude, unpublished).[30] Further molecular analysis will probably provide additional clues on how to extend longevity independently of the organism's reproductive ability.

## ACKNOWLEDGMENTS

I would like to thank David W. Walker and Ted Brummel for comments and suggestions on this manuscript and members of Pr. Seymour Benzer's lab for discussions. Aging research in the author's laboratory is supported by a new investigator grant from Queen's University.
